# Supplementary material for: Association between first-trimester cell-free fetal DNA levels and the risk of preterm birth and low birth weight: A propensity score-matched cohort study
Source: PLoS One. 2026 May 11;21(5):e0349018. doi: 10.1371/journal.pone.0349018 (PMC13160320; doi:10.1371/journal.pone.0349018)
Supplement: Attachment — Submitted filename: Response to Reviewers.docx [file pone.0349018.s003.docx]

**Response Letter**

Dear editors and reviewers,

We deeply appreciate your valuable suggestions and comments, which enable us to further improve our paper. We have studied the comments carefully and have made our best efforts to revise the entire paper as suggested by the editor and reviewers. We are very pleased that our paper can be revised, some **detailed responses** are as follows.

**Journal Requirements:**

1.Please ensure that your manuscript meets PLOS ONE's style requirements, including those for file naming. The PLOS ONE style templates can be found at

https://journals.plos.org/plosone/s/file?id=wjVg/PLOSOne_formatting_sample_main_body.pdf and

[https://journals.plos.org/plosone/s/file?id=ba62/PLOSOne_formatting_sample_title_authors_affiliations.pdf](https://track.editorialmanager.com/CL0/https:%2F%2Fjournals.plos.org%2Fplosone%2Fs%2Ffile%3Fid=ba62%2FPLOSOne_formatting_sample_title_authors_affiliations.pdf/1/010f019ab38e29f4-b918e807-0bd2-4425-9acd-4f5d01ca0bb1-000000/YlVqxIrrYbwGVLi3d2nt2G2rbpDdPI0QSJgWHiQMkco=238)

**Response :** Thank you for the reminder, we have updated the manuscript to comply with PLOS ONE's style requirements.

2. Please amend the manuscript submission data (via Edit Submission) to include author “Xiaosa Wang”.

**Response :** Thank you for your message. We have updated the submission data via the "Edit Submission" feature to include author Xiaosa Wang as requested.

3. Please amend your authorship list in your manuscript file to include author “Xiasa Wang”.

**Response :** Thank you for your guidance. We have amended the authorship list in our manuscript file to include Xiasa Wang and have uploaded the revised version to the submission system.

4. If the reviewer comments include a recommendation to cite specific previously published works, please review and evaluate these publications to determine whether they are relevant and should be cited. There is no requirement to cite these works unless the editor has indicated otherwise.

**Response :** Thank you for the note. We confirm that no additional citation requests were made by the reviewers.

**To Additional Editor:**

1. Introduction: the rationale of the study makes preemptive assumptions which must be avoided.  The rationale should specify the aim of the study with the approach to solving the challenge with a risk based stratified approach.

**Response :** We sincerely thank you for the valuable comment regarding the rationale of our study. In response to the concern about preemptive assumptions in the introduction, we have thoroughly revised the third and fourth paragraphs of the Introduction section. The modifications include reframing the study rationale around the existing uncertainty regarding first-trimester cell-free fetal DNA (cffDNA) as a biomarker for risk stratification, clearly stating the study aim to address this gap, and explicitly describing the use of cffDNA quartiles and multiple adjusted regression models as part of a risk-based analytical approach. All changes have been highlighted in red in the manuscript text for easy reference.

2. The methods do not comply and must be corrected as follows:

- The molecular methods used for cffDNA must be included.
- The is a statement made about date of accessing data.  Was data used from another study or source, if yes please specify.

- Please specify the reason for the removal of informed consent as this goes against the declaration of helinski Please provide a valid justification.  Failure would result in rejection of the manuscript.

- I suggest that a methodology map be created to explain the detailed statistical analysis done per a question that is trying to be answered.

**Response : (1)** We thank you for raising this important point. In response to the comment regarding molecular methods for cffDNA quantification, we have now added a detailed description in the Data Collection subsection of the Methods. Specifically, we have included the following information marked in red: "Cell-free fetal DNA (cffDNA) levels, expressed as fetal fraction (%), were quantitatively measured through clinical non-invasive prenatal testing (NIPT). The analysis was performed following standard protocols: maternal peripheral blood samples were collected, maternal plasma separation, DNA extraction, next-generation sequencing on the Illumina platform, and bioinformatic quantification of the fetal DNA fraction." This addition provides the technical details of cffDNA measurement.

**(2)** We thank you for this important question regarding data access. The data used in this study were original clinical data retrospectively collected from the electronic medical records of Shaoxing Maternal and Child Health Care Hospital for the specific purpose of this research. They are not sourced from an external database or a previously published study. The date “1 May 2025” mentioned in the manuscript was intended to indicate the point at which our final study dataset was locked for analysis, not the date of accessing an external source.

**(3)** We sincerely thank you for raising this critical ethical issue. The waiver of informed consent for this retrospective study was formally approved by our Institutional Review Board (Approval No: [IRB-AF-023-01.5]) as it aligns with international ethical guidelines under the following justifications: 1. the research involves no more than minimal risk as it exclusively utilizes fully de-identified clinical data; 2. obtaining individual consent is impracticable given the large-scale historical cohort (n=10,345) where participant re-contact is impossible; 3. the waiver does not adversely affect the rights or welfare of participants; 4. robust privacy protection measures were implemented, including permanent deletion of all direct identifiers and secure data encryption. For your convenience, we have uploaded the official ethical approval documents (both Chinese and English versions) which explicitly state the waiver of informed consent, to the submission system. We are confident that our approach fully complies with all regulatory requirements.

**(4)** We thank you for this valuable suggestion. In response, we have created a comprehensive methodology flowchart that visually outlines the entire analytical plan, from the initial cohort selection and propensity score matching to the specific statistical analysis applied to each research question. This figure has now been included as Figure 1 in the main manuscript. We believe it greatly enhances the clarity of our statistical approach.

3. In the results section  more graphical presentation of data is required.

**Response :** We sincerely thank you for the suggestion to enhance the graphical presentation of our results. In direct response to this comment, we have now created additional figures to visually summarize the key findings of our study, bringing the total to six figures. Regarding the more detailed results in Tables 1-4, which present the baseline characteristics and the comprehensive univariate/multivariable logistic regression outputs before and after propensity score matching, we have deliberately kept these in a tabular format. We believe that for these specific sets of data, tables remain the clearest and most conventional method to display a large number of variables and statistical parameters, ensuring precision and facilitating easy comparison for the reader. Converting these extensive tables into figures would risk creating visual complexity that could obscure the detailed findings. We hope that the current structure of six figures and four tables provides an optimal balance, offering both high-level visual insight and the necessary granularity for in-depth scientific scrutiny. We hope the revised manuscript now meets with your approval.

We are very grateful for your insightful comments and suggestions, which are extremely valuable and helpful in improving the quality of our manuscript.

Sincerely yours,

Xiaosa Wang, Hui Shao, Junjiang Wu, Guofeng Yin
