# Supplementary material for: Association between first-trimester cell-free fetal DNA levels and the risk of preterm birth and low birth weight: A propensity score-matched cohort study
Source: PLoS One. 2026 May 11;21(5):e0349018. doi: 10.1371/journal.pone.0349018 (PMC13160320; doi:10.1371/journal.pone.0349018)
Supplement: Attachment — Submitted filename: Response_to_Reviewers_auresp_2.docx [file pone.0349018.s004.docx]

**Response Letter**

Dear editors and reviewers,

We deeply appreciate your valuable suggestions and comments, which enable us to further improve our paper. We have studied the comments carefully and have made our best efforts to revise the entire paper as suggested by the editor and reviewers. We are very pleased that our paper can be revised, some **detailed responses** are as follows.

**Journal Requirements:**

1. If the reviewer comments include a recommendation to cite specific previously published works, please review and evaluate these publications to determine whether they are relevant and should be cited. There is no requirement to cite these works unless the editor has indicated otherwise.

**Response :** Thank you for this important reminder regarding journal requirements. We have carefully reviewed the publications mentioned in the reviewer's comments and have evaluated their relevance to our study. Where appropriate and directly relevant to our methodology, discussion, or interpretation of findings, we have cited these works in the revised manuscript. Specifically, we have incorporated the study by Becking et al. (2024) in the Discussion section to contextualize the clinical utility and predictive value of fetal fraction, and we have added references to support our methodological choices, including the definition of advanced maternal age [12], obesity classification [13]. All citations have been carefully evaluated to ensure they meaningfully contribute to the scientific rigor and transparency of our manuscript. We confirm that no works have been cited solely in response to reviewer suggestions without proper consideration of their relevance. Thank you for your guidance.

2. Please review your reference list to ensure that it is complete and correct. If you have cited papers that have been retracted, please include the rationale for doing so in the manuscript text, or remove these references and replace them with relevant current references. Any changes to the reference list should be mentioned in the rebuttal letter that accompanies your revised manuscript. If you need to cite a retracted article, indicate the article’s retracted status in the References list and also include a citation and full reference for the retraction notice.

**Response :** Thank you for this important instruction regarding the reference list. We have carefully reviewed the entire reference list to ensure its completeness and accuracy. We confirm that all references cited in our manuscript are relevant, current, and appropriately support the statements made in the text. We have verified that none of the cited papers have been retracted. Additionally, we have checked that all references are formatted consistently according to the journal's guidelines and that no duplicate or irrelevant citations are included. Any changes made to the reference list during the revision process, including the addition of new references [12,13,25] to address reviewer comments, have been clearly documented in our rebuttal letter and highlighted in the revised manuscript. We believe the reference list is now complete and correct. Thank you for your guidance.

**To Additional Editor:**

For the manuscript to be accepted all reviewers' comments must be addressed with a clear rebuttal letter that tracks all changes in the manuscript.

**Response :** Thank you for your clear guidance regarding the revision requirements. We confirm that we have carefully addressed all reviewers' comments in this revised manuscript. A point-by-point rebuttal letter has been prepared, detailing how each comment has been responded to and explicitly tracking all corresponding changes made in the manuscript. All revisions have been clearly highlighted in red in the manuscript for your easy reference. We believe that the manuscript has been substantially improved through this process and now meets the journal's standards for publication. We appreciate the time and effort of the editors and reviewers in providing constructive feedback, and we hope that the revised version is now acceptable for publication. Thank you for your consideration.

**To Reviewer #1:**

This revised manuscript to me is scientifically sound. It shows the association between first-trimester fetal fraction and preterm birth and low birth weight. It adds to the body of work on this subject. I have some minor issues that I believe should be addressed.
- In the Objective in the Abstract add 'first trimester'

**Response :** Thank you for your insightful suggestion. We have revised the Objective in the Abstract to specify that the investigation focuses on cffDNA concentration measured in the first trimester. The revised sentence now reads: "To investigate the association between the concentration of cell-free fetal DNA (cffDNA) in the first trimester and the risks of preterm birth (PTB) and low birth weight (LBW) in a large cohort." We believe this modification enhances the clarity and precision of our study objective.

- For readability, I suggest not to put all the OR's/aOR's and 95%-CI's in the text of the Result section. They are already presented in the tables.

**Response :** Thank you very much for your thoughtful and constructive suggestion regarding the readability of the Results section. We completely agree that the text should be concise and avoid unnecessary repetition of numerical data that are already well-presented in the tables. In response to your comment, we have carefully revised the manuscript. Specifically, for sections where the odds ratios and confidence intervals were fully detailed in the corresponding tables (e.g., the general logistic regression results in Table 3 and 4), we have streamlined the text to focus on the direction, significance, and patterns of the associations, rather than listing every numerical value. However, for sections where the results were referenced alongside figures that do not display the exact numerical data—such as the hierarchical logistic regression models (Fig 3) and the threshold effect analysis (Fig 6)—we retained the specific ORs and CIs in the text to ensure clarity and precision for the reader. We believe this balanced approach enhances readability while maintaining the scientific rigor and completeness of our findings.

We hope this revision aligns with your expectations. Thank you again for your valuable input, which has helped improve the quality of our manuscript.

- In the Discussion section, could the authors provide more insight from a clinical point of view how fetal fraction could be a useful biomarker? Indeed, associations with adverse pregnancy outcomes have been shown, but does it really help to predict adverse outcomes and change individual pregnancy care? Possibly comment hereon also in light of a publication by Becking et al. on the predictive value of fetal fraction (Becking et al. BJOG, 2024; https://doi.org/10.1111/1471-0528.17978)

**Response :** Thank you for this insightful and thought-provoking comment, which challenges us to critically evaluate the clinical translation of our findings. We agree that demonstrating statistical associations is distinct from establishing clinical utility for individual risk prediction and pregnancy care. In response to your suggestion, we have expanded the Discussion section to address this important issue directly, incorporating the recent work by Becking et al. (2024) on the predictive value of fetal fraction.

Specifically, we have added the following paragraph: "From a clinical perspective, an important question is whether these robust associations translate into meaningful improvements in risk prediction and individual pregnancy care. Becking et al. [25] recently addressed this question in a large nationwide cohort of 56,110 pregnancies, demonstrating that while fetal fraction shows statistically significant associations with adverse outcomes, its added prognostic value beyond established clinical risk factors is limited. In their study, adding fetal fraction to prediction models resulted in only modest improvements in discrimination, with increases in the area under the curve ranging from 0.01 to 0.02 and small but significant integrated discrimination improvement indices, leading the authors to conclude that fetal fraction has 'statistically significant but limited prognostic value'. These findings contextualize our own results: the protective associations we observed, while consistent and biologically plausible, may not be sufficient to use cffDNA as a standalone predictor in clinical practice. However, because fetal fraction is routinely measured as a quality parameter in non-invasive prenatal testing without additional cost, it could still contribute to broader risk stratification strategies when integrated with other clinical parameters."

We believe this addition provides a balanced and clinically grounded perspective, acknowledging both the potential and the limitations of cffDNA as a biomarker for adverse pregnancy outcomes. Thank you again for your valuable input, which has helped strengthen the clinical relevance and academic rigor of our manuscript.

- Last, LWB without correcting for gestational age has little clinical meaning, this should be mentioned as a limitation of the study. Or are gestational-age corrected birthweights available?

**Response :** Thank you for this important methodological comment. We acknowledge that low birth weight (LBW) is a composite outcome influenced by both gestational age at delivery and fetal growth restriction. In our study, we did not have access to population-based, gestational-age-adjusted birth weight percentiles (e.g., small for gestational age, SGA) to distinguish between these two pathways. Therefore, as you correctly pointed out, the interpretation of our LBW findings is limited, as they may reflect effects driven primarily by preterm birth rather than impaired fetal growth. We have now added this point as a limitation in the Discussion section: "Additionally, LBW is a composite outcome influenced by both gestational age at delivery and fetal growth restriction. Our analysis did not adjust for gestational age (e.g., using small for gestational age [SGA] as an outcome), which limits the clinical interpretation of the LBW findings, as the observed protective effects may be primarily driven by the prevention of preterm birth rather than enhanced fetal growth. Future studies should incorporate gestational-age-corrected birthweight measures to disentangle these pathways." This addition ensures that readers interpret the LBW results with appropriate caution. Thank you again for your valuable insight, which has helped strengthen the rigor and transparency of our manuscript.

**To Reviewer #2:**

FINDING ON THE AUTHOR'S RESPONSE TO THE 1ST REVIEWER'S CONCERNS.
All comments have been adequately addressed except for the method for molecular description

1st Reviewer comments: Require explicit molecular/technical description of cffDNA measurement.
My Findings: The current submission has substantially addressed the comments but remains borderline. The platform is named, but key technical details such as sequencing depth, library prep method, and fetal fraction algorithm (e.g., SNP-based vs. read-count) are missing.
In my opinion, if NIPT is a routine clinical test, the level of detail might not be necessary. However, clarifying sentences must be added. e.g., “Fetal fraction was calculated using a validated read-count–based algorithm routinely applied in clinical NIPT reporting”.

**Response :** Thank you for your thorough evaluation and for providing specific guidance on the methodological description of cffDNA measurement. We agree that explicit technical details are important for reproducibility and scientific rigor, even when describing a routine clinical test. In response to your comment, we have now revised the Methods section to include a more detailed description of the cffDNA measurement process.

The revised section in the Methods section of the manuscript now reads as follows: " Cell-free fetal DNA (cffDNA) levels, expressed as fetal fraction (%), were quantitatively measured through clinical non-invasive prenatal testing (NIPT). The analysis was performed following standard protocols: maternal peripheral blood samples were collected, maternal plasma separation, DNA extraction, **library preparation using the Illumina TruSeq Nano DNA Library Prep Kit,** next-generation sequencing on the Illumina platform **with a median sequencing depth of approximately 0.2X (10 million reads per sample),** and bioinformatic quantification of the fetal DNA fraction. **Fetal fraction was calculated using a validated algorithm that combines two approaches depending on fetal sex: for male fetuses, the algorithm is based on the relative coverage of chromosome Y sequences; for female fetuses, a neural network model trained on autosomal read count patterns and fragment size distributions is used to estimate fetal fraction. This algorithm is routinely applied in clinical NIPT reporting in our laboratory. A single, consistent analytical pipeline was maintained throughout the entire study period from June 2023 to June 2025, with no changes to the sequencing platform, library preparation protocol, or fetal fraction estimation algorithm. All samples were processed and analyzed using the same standardized protocols in our clinical laboratory. Quality control thresholds were applied to ensure reliable cffDNA measurement: only samples with a minimum sequencing depth of 10 million reads and a fetal fraction value ≥4% were included in the final analysis, as lower fetal fractions are associated with increased risk of test failure and may reflect poor sample quality or underlying placental abnormalities. Samples failing to meet these quality criteria were excluded from the study cohort.** "

We believe these additions provide the necessary technical clarity while maintaining an appropriate level of detail for a clinically oriented study. Thank you again for your constructive suggestion, which has helped strengthen the methodological transparency of our manuscript.

REVIEWER REPORT
I have some concerns with the current submission. Below is my report with major and minor concerns addressed separately

General Assessment
This manuscript reports a large retrospective cohort study examining the association between first-trimester cell-free fetal DNA (cffDNA) levels and the risks of preterm birth (PTB) and low birth weight (LBW), using propensity score matching and multivariable regression analyses. The study addresses an area of ongoing debate, particularly regarding the directionality of cffDNA associations with adverse pregnancy outcomes.
Overall, the study is methodologically sound, ethically compliant, and clearly presented. The conclusions are largely supported by the data. However, several conceptual and methodological clarifications are required to strengthen interpretability and reproducibility, particularly regarding cohort selection, cffDNA measurement, and interpretation of subgroup/threshold analyses.

**Response :** Thank you very much for your thoughtful and constructive feedback on our manuscript. We greatly appreciate your positive assessment of our work, particularly your recognition that the study is methodologically sound, ethically compliant, and clearly presented, and that the conclusions are largely supported by the data. We also thank you for your insightful suggestions regarding the need for further clarifications on cohort selection, cffDNA measurement, and the interpretation of subgroup and threshold analyses. These are important points that will help strengthen the interpretability and reproducibility of our study. We will carefully address each of these comments in the revised version of the manuscript and provide a detailed point-by-point response. We believe that incorporating your suggestions will significantly improve the quality and clarity of our work. Thank you again for your time and expertise in reviewing our manuscript.

Major Strengths
1. Large sample size and statistical power
The cohort of over 10,000 singleton pregnancies provides robust power to detect modest associations and to conduct stratified analyses.
2. Appropriate use of propensity score matching (PSM)
The authors appropriately apply PSM to address confounding in this observational study and demonstrate adequate post-matching balance using standardized mean differences.
3. Outcome-specific analysis (PTB vs LBW)
The distinction between PTB and LBW is well justified biologically and analytically, and the differential association patterns are a notable strength.
4. Ethical transparency
Institutional approval, waiver of informed consent, and anonymisation procedures are clearly stated and acceptable for retrospective research.

**Response :** Thank you very much for your positive and encouraging feedback on our manuscript. We are sincerely grateful for your recognition of the major strengths of our study, including the large sample size and statistical power, the appropriate use of propensity score matching to address confounding, the biologically and analytically well-justified distinction between preterm birth and low birth weight outcomes, and the clear ethical transparency in our reporting. Your acknowledgment of these methodological strengths is highly encouraging to our team. We appreciate the time and expertise you have dedicated to reviewing our work.

Major Scientific Concerns
1. Cohort Selection and Potential Selection Bias (Important)
It appears that cffDNA measurements were derived from routine clinical non-invasive prenatal testing (NIPT). However, it is not clearly stated whether:
1. All singleton pregnancies during the study period underwent NIPT, or
2. The cohort represents a selected subgroup (e.g. advanced maternal age, higher socioeconomic status, or high-risk pregnancies).
This point is critical because NIPT uptake is not random and may introduce selection bias that could influence both cffDNA levels and pregnancy outcomes.
Suggestions:
Explicitly clarify the NIPT inclusion criteria and discuss potential selection bias and its impact on generalisability in the Discussion.

**Response :** Thank you for raising this important point regarding potential selection bias and the generalizability of our findings. We agree that clarifying the NIPT inclusion criteria and discussing their implications is essential for the interpretability of our study.

In response to your comment, we have now explicitly stated the NIPT indication criteria in the Methods section. Specifically, we have added the following description: "In our institution, non-invasive prenatal testing (NIPT) was offered to pregnant women meeting specific clinical criteria during the study period. These indications included: (1) women with first-trimester serum screening results indicating a risk for common fetal chromosomal aneuploidies between the high-risk cutoff and 1/1000; (2) women with contraindications to invasive prenatal diagnosis (e.g., threatened miscarriage, fever, bleeding tendencies, active chronic infectious diseases, or Rh-negative blood type); and (3) women who voluntarily requested risk assessment for trisomy 21, trisomy 18, and trisomy 13. Consequently, the study cohort represents a selected population of pregnant women who underwent NIPT based on these indications, rather than an unselected general obstetric population."

Additionally, we have expanded the final paragraph of the Discussion section to address the implications of this selection. The revised limitation now reads: "Furthermore, as described in the Methods section, NIPT in our institution was offered to women with specific indications, including intermediate-risk results on first-trimester serum screening, contraindications to invasive procedures, or voluntary requests for aneuploidy risk assessment. Therefore, our cohort represents a selected subgroup of the pregnant population rather than an unselected general obstetric cohort. This selection may introduce bias, as women undergoing NIPT based on these indications may have different baseline characteristics and risk profiles compared to the broader population of pregnant women. For instance, women with intermediate-risk serum screening results may have distinct placental or biochemical profiles that could influence both cffDNA levels and pregnancy outcomes. Consequently, the observed associations between cffDNA and PTB/LBW may not be directly generalizable to lower-risk populations, to settings with different NIPT utilization policies, or to populations where NIPT is offered as a first-tier screening test to all pregnant women. Future studies in unselected cohorts or diverse healthcare settings with varying NIPT access criteria are warranted to validate our findings and assess their external validity."

We believe these additions appropriately address your concern and provide readers with the necessary context to interpret our findings with appropriate caution. Thank you again for your insightful suggestion.

2. cffDNA Measurement and Reproducibility (Moderate)
While the manuscript now includes a general description of the NIPT workflow (plasma separation, DNA extraction, NGS, Illumina platform), key technical details relevant to reproducibility remain unclear:
1. How was fetal fraction calculated (e.g. read-count, SNP-based, proprietary algorithm)?
2. Was a single analytical pipeline used throughout the study period?
3. Were quality thresholds applied for inclusion of cffDNA values?
Although full proprietary details may not be required, some clarification is necessary for scientific transparency.
Suggestions:
Add a brief statement describing the fetal fraction estimation approach and confirm analytical consistency across samples.

**Response :** Thank you for your thoughtful comments regarding the cffDNA measurement and reproducibility. In response to your suggestions, we have revised the Methods section to provide additional technical clarity. Specifically, we have now explicitly described the fetal fraction estimation approach: for male fetuses, the algorithm is based on relative coverage of chromosome Y sequences; for female fetuses, a neural network model trained on autosomal read count patterns and fragment size distributions is used. We have also confirmed that a single, consistent analytical pipeline was maintained throughout the entire study period (June 2023 to June 2025), with no changes to the sequencing platform, library preparation protocol, or fetal fraction estimation algorithm. Additionally, we have specified the quality control thresholds applied for inclusion: only samples with a minimum sequencing depth of 10 million reads and a fetal fraction value ≥4% were included in the final analysis. We believe these additions address your concerns regarding scientific transparency and reproducibility. All revisions have been highlighted in red in the manuscript for your easy reference. Thank you again for your valuable feedback.

3. Interpretation of Subgroup and Threshold Analyses (Important)
The manuscript includes extensive subgroup and threshold analyses. However:
• It is unclear whether these analyses were pre-specified or exploratory.
• Multiple comparisons increase the risk of type I error.
• Threshold effects may be data-driven.
While these analyses are interesting, their inferential weight should be limited. I presumed the analysis is exploratory; thus, concern about overstating findings should be addressed.
Suggestions:
Explicitly state that subgroup and threshold analyses are exploratory and interpret findings cautiously in the Discussion or suggest a study in an independent cohort.

**Response :** Thank you for your important methodological comment regarding the interpretation of our subgroup and threshold analyses. We fully agree that the exploratory nature of these analyses, the potential for type I error due to multiple comparisons, and the data-driven selection of thresholds warrant appropriate caution in interpretation. In response to your suggestion, we have added the following statement in the Statistical analysis section: "All subgroup analyses and threshold effect analyses were exploratory in nature and were not pre-specified in the study protocol. Given the multiple comparisons performed, these findings should be interpreted with caution and considered hypothesis-generating rather than confirmatory." Additionally, in the Discussion section, we have included the following paragraph in the limitations: "Finally, the subgroup and threshold analyses presented in this study were exploratory and not pre-specified. The multiple comparisons performed increase the risk of type I errors, and the data-driven nature of threshold selection may limit the reproducibility of the identified cutoff values. Therefore, these findings should be considered hypothesis-generating, and future studies in independent cohorts are needed to validate the observed patterns and interactions." Both additions have been highlighted in red in the revised manuscript for your easy reference. We believe these revisions appropriately address your concern and provide readers with the necessary context to interpret our findings with appropriate caution. Thank you again for your insightful feedback.

4. Biological Interpretation of “Protective” cffDNA Effects (Conceptual)
The finding that higher first-trimester cffDNA levels are associated with lower PTB risk contrasts with traditional interpretations of cffDNA as a marker of placental stress or injury. While the authors briefly allude to placental mass or turnover, the biological interpretation remains underdeveloped.
Critically, in the Discussion section (where this interpretation should be expanded):There is no clear paragraph that:
1. reconciles the inverse association with placental biology
2. distinguishes pathological cffDNA elevation from physiological placental mass/turnover
3. explains why higher early fetal fraction could reflect healthier placentation
Suggestion:
Expand the Discussion to more clearly distinguish between:
1. pathological cffDNA elevation due to placental damage, and
2. physiologically higher cffDNA reflecting placental mass or healthy trophoblast turnover.
**Response :** Thank you for your insightful comment regarding the biological interpretation of the protective cffDNA effects. We agree that reconciling our findings with traditional views of cffDNA as a marker of placental stress requires clearer conceptual framing. In response, we have expanded the Discussion section to explicitly distinguish between pathological cffDNA elevation due to placental damage and physiologically higher cffDNA reflecting healthy placental mass and trophoblast turnover. The revised paragraph now reads: "At first glance, this finding may appear to contrast with traditional interpretations of cffDNA as a marker of placental stress or injury, where elevated levels might be expected to correlate with adverse outcomes. However, this apparent paradox can be reconciled by distinguishing between two distinct biological contexts that lead to elevated cffDNA. On one hand, pathological elevations in cffDNA can occur as a result of placental damage, inflammation, or apoptosis secondary to hypoxic or oxidative stress—situations where the placenta is under duress and at increased risk of dysfunction. On the other hand, physiologically higher cffDNA levels in the first trimester may simply reflect greater placental mass, more robust trophoblast invasion, and healthy, regulated trophoblast turnover—all indicators of optimal placental development and function. Our findings align with this latter interpretation: in a generally low-risk population without overt placental pathology, higher cffDNA likely represents better placentation rather than incipient injury. This distinction is critical: the same biomarker can have different implications depending on the clinical context, timing of measurement, and underlying placental biology." This addition clarifies why higher first-trimester cffDNA can be associated with reduced PTB risk and provides a more nuanced biological framework for interpreting our findings. The revised text has been highlighted in red in the manuscript for your easy reference. Thank you again for your valuable suggestion.

5.Fixed threshold for maternal age and BMI
The manuscript categorises maternal age and BMI using fixed thresholds; however, the rationale for selecting ≥35 years for age and ≥30 kg/m² for BMI is not explicitly stated. As these variables are central to the propensity score model, a brief justification based on clinical guidelines or prior literature would improve transparency and reproducibility.

**Response :** Thank you for your comment regarding the fixed thresholds used for maternal age and BMI. We agree that providing a clear rationale for these cutoffs is important for transparency and reproducibility, particularly as these variables are central to our propensity score matching model. In response to your suggestion, we have added a brief justification in the Methods section (Data collection subsection) to clarify the basis for these thresholds. The following sentences have been included: "Maternal age was dichotomized at ≥35 years based on the widely accepted definition of advanced maternal age, which is associated with increased risks of adverse pregnancy outcomes including preterm birth and low birth weight [12]. Pre-delivery body mass index (BMI) was categorized using a threshold of ≥30 kg/m², consistent with the definition of obesity as a well-established risk factor for pregnancy complications [13]." Appropriate references [12,13] have been cited to support these definitions. We believe this addition addresses your concern and enhances the methodological transparency of our manuscript. The revised text has been highlighted in red for your easy reference. Thank you again for your valuable feedback.

Minor Issues and Editorial Suggestions

1. Causal language
Terms such as “protective effect” should be replaced or clearly contextualised as “inverse association” to avoid causal implication.

**Response :** We thank you for the insightful comment regarding the use of causal language. We agree that terms such as “protective effect” should be avoided in observational studies to prevent unintended causal implications. In response, we have carefully reviewed the manuscript and replaced all instances of “protective effect” with “inverse association” throughout the text. To facilitate the review process, all revised terms have been highlighted in red in the marked version of the manuscript. We believe this revision more accurately reflects the observational nature of our findings and enhances the scientific rigor of our wording. Thank you again for this valuable suggestion.

2. Results–Discussion boundary
Some interpretive language appears in the Results section; tightening this separation would improve clarity.

**Response :** Thank you for your valuable comment regarding the boundary between Results and Discussion. We agree that maintaining a clear separation between objective findings and interpretive language enhances the clarity and scientific rigor of the manuscript.

In response to your suggestion, we have carefully reviewed the Results section and made the following revisions:

From the "Univariate and Multivariate Logistic Regression Analysis of CffDNA and PTB/LBW" subsection, we have deleted the concluding sentence that contained interpretive summary: "These findings demonstrate a consistent inverse relationship between elevated cffDNA levels and reduced preterm birth risk across all quartiles, while for low birth weight, significant inverse associations were observed specifically in the highest cffDNA quartile."

From the "Threshold Effect Analysis" subsection, we have deleted the final summary paragraph: "In summary, a significant threshold effect of cffDNA on PTB was consistently observed both before and after PSM. For LBW, a significant threshold effect was present only before PSM and was not maintained after matching."

The revised Results section now focuses exclusively on the objective presentation of data in these subsections, with all interpretive commentary appropriately retained in the Discussion section. We believe these revisions have appropriately addressed your concern and improved the clarity of our manuscript. Thank you again for your thoughtful feedback.

3. Author name consistency
Ensure consistent spelling of author names across the manuscript and submission metadata.

**Response :** Thank you for pointing this out. We have thoroughly reviewed the manuscript and verified that all author names are spelled consistently throughout the main text, title page, and submission metadata. Any discrepancies have been corrected accordingly.

4. Justification of PSM parameters
A brief justification for the chosen 1:3 matching ratio would be helpful.

**Response :** Thank you for your comment regarding the justification of our propensity score matching parameters. In response to your suggestion, we have added a brief explanation for the chosen 1:3 matching ratio in the Statistical analysis section of the Methods. The following sentence has been included: "The 1:3 matching ratio was selected to maximize statistical power while maintaining adequate balance between groups, as matching more controls to each case can increase the precision of treatment effect estimates without substantially increasing bias, particularly when the number of available controls is sufficient." We believe this addition enhances the transparency and reproducibility of our analytical approach. The revised text has been highlighted in red in the manuscript for your easy reference. Thank you again for your valuable feedback.

We are very grateful for your insightful comments and suggestions, which are extremely valuable and helpful in improving the quality of our manuscript.

Sincerely yours,

Xiaosa Wang, Hui Shao, Junjiang Wu, Guofeng Yin
